# The effects of intermittent theta burst stimulation of the unilateral cerebellar hemisphere on swallowing-related brain regions in healthy subjects

**DOI:** 10.3389/fnhum.2023.1100320

**Published:** 2023-03-30

**Authors:** Bingyan Wang, Hui Sun, Xiaona Pan, Wenshuai Ma, Linghui Dong, Qiang Wang, Pingping Meng

**Affiliations:** ^1^Department of Rehabilitation Medicine, Affiliated Hospital of Qingdao University, Qingdao, China; ^2^Department of Radiology, Affiliated Hospital of Qingdao University, Qingdao, China

**Keywords:** swallowing, cerebellum, intermittent theta burst stimulation (iTBS), resting-state functional magnetic resonance imaging (rs-fMRI), fractional amplitude of low-frequency fluctuation (fALFF)

## Abstract

**Objective:**

We aimed to investigate the effects and mechanisms of swallowing-related brain regions using resting-state functional magnetic resonance imaging (rs-fMRI) in healthy subjects who underwent intermittent theta burst stimulation (iTBS) on dominant or non-dominant cerebellar hemispheres.

**Methods:**

Thirty-nine healthy subjects were randomized into three groups that completed different iTBS protocols (dominant cerebellum group, non-dominant cerebellum group and sham group). Before iTBS, the resting motor threshold (rMT) was measured by single-pulse transcranial magnetic stimulation (sTMS) on the cerebellar representation of the suprahyoid muscles, and the dominant cerebellar hemisphere for swallowing was determined. Forty-eight hours after elution, iTBS protocols were completed: in the dominant cerebellum group, iTBS was administered to the dominant cerebellar hemisphere, and the non-dominant cerebellar hemisphere was given sham stimulation; in the non-dominant cerebellum group, iTBS was administered to the non-dominant cerebellar hemisphere, and sham stimulation was delivered to the dominant cerebellar hemisphere; in the sham group, sham stimulation was applied to the cerebellum bilaterally. Rs-fMRI was performed before and after iTBS stimulation to observe changes in the fractional amplitude of low-frequency fluctuation (fALFF) in the whole brain.

**Results:**

Compared with baseline, the dominant cerebellum group showed increased fALFF in the ipsilateral cerebellum, and decreased fALFF in the ipsilateral middle temporal gyrus and contralateral precuneus after iTBS; the iTBS of the non-dominant cerebellum group induced increased fALFF in the ipsilateral superior frontal gyrus, the calcarine fissure and the surrounding cortex, and the contralateral inferior parietal lobule; and in the sham group, there was no significant difference in fALFF. Exploring the effects induced by iTBS among groups, the dominant cerebellum group showed decreased fALFF in the contralateral calcarine fissure, and surrounding cortex compared with the sham group.

**Conclusion:**

Intermittent theta burst stimulation of the dominant cerebellar hemisphere for swallowing excited the ipsilateral cerebellum, and stimulation of the non-dominant cerebellar hemisphere increased the spontaneous neural activity of multiple cerebrocortical areas related to swallowing. In conclusion, regardless of which side of the cerebellum is stimulated, iTBS can facilitate part of the brain neural network related to swallowing. Our findings provide supporting evidence that cerebellar iTBS can be used as a potential method to modulate human swallowing movement.

## 1. Introduction

Dysphagia is a common dysfunction in stroke patients, with a high incidence of 45.06% (Rofes et al., 2018). Poststroke dysphagia not only has a great impact on the quality of life of patients, but also leads to malnutrition, electrolyte disturbance, and even aspiration (Altman et al., 2010; Clavé et al., 2012). Aspiration can cause complications, such as aspiration pneumonia, which greatly delay the recovery of stroke and even endanger patients’ lives. In clinical practice, the commonly used traditional treatments for dysphagia mainly include volume and texture modification, behavioral compensation (chin tuck, head tilt, head turn, etc.), sensory and sensorimotor training, swallowing auxiliary methods (supraglottic swallow, super-supraglottic swallow, Mendelsohn maneuver, etc.), neuromuscular electrical stimulation, acupuncture and others. Although traditional methods have been widely used in the clinic, their efficacy in dysphagia has not been confirmed by sufficient evidence-based medical proof (Vose et al., 2014; Bath et al., 2018).

Repetitive transcranial magnetic stimulation (rTMS) is a non-invasive brain stimulation technique that can directly stimulate brain tissues. Because of its advantages of painlessness, convenient and rapid operation, few adverse reactions and relative safety, rTMS has been used in research by many scholars to improve the efficacy in dysphagia. Theta burst stimulation (TBS) is a patterned rTMS protocol that simulates the pulse release frequency of the human hippocampal gyrus (Huang et al., 2005), and can effectively regulate the excitability of the cerebral swallowing cortex (Lin et al., 2017). Compared with conventional rTMS, it has the characteristics of high frequency, low intensity, and short duration (Di Lazzaro et al., 2011; Huang et al., 2011). There are two common paradigms: intermittent theta burst stimulation (iTBS), which has a facilitatory effect on the stimulated area, and continuous theta burst stimulation (cTBS), which can inhibit the target area (Chung et al., 2016).

Currently, the stimulation target of rTMS studies in dysphagia mainly focuses on the cerebral cortex. Whether stimulating the ipsilateral hemisphere, the contralateral hemisphere or the bilateral hemisphere, the studies all have shown curative effects (Khedr et al., 2009; Lim et al., 2014; Park et al., 2017). However, stimulating the cerebral cortex has some limitations, such as the complex operation of locating the target (Sasegbon et al., 2019) and the risk of seizures (Rossi et al., 2009). In addition, skull defects limit the application of rTMS in patients after cerebral hemorrhage operations. All these factors have prompted researchers to explore new stimulation targets. As studies have shown that the cerebellum also plays a role in human swallowing (Zald and Pardo, 1999; Mosier and Bereznaya, 2001), the exploration of cerebellar rTMS on dysphagia is gradually emerging. Jayasekeran et al. (2011) applied single-pulse TMS to the cerebellum of healthy subjects and found that it can induce motor evoked potentials (MEPs) of pharyngeal muscles, demonstrating for the first time that cerebellar TMS can modulate human swallowing movement. Subsequently, Sasegbon et al. (2019) confirmed that unilateral cerebellar rTMS can excite bilateral cerebral hemispheres using a cortical virtual lesion model. In 2021, a randomized controlled trial of patients with poststroke dysphagia conducted by Zhong et al. (2021) showed the safety and efficacy of cerebellar rTMS, but its concrete mechanism is still unclear.

Similar to the phenomenon of cortical hemispheric asymmetry during swallowing, the control of the cerebellum over swallowing also shows laterality. An event-related functional magnetic resonance imaging (fMRI) study by Suzuki et al. (2003) found that bilateral cerebellar hemispheres were asymmetrically activated during swallowing, and Malandraki et al. (2009) further verified this phenomenon. To date, no studies have explored the differences in regulating swallowing function between stimulating the dominant cerebellar hemisphere for swallowing, and the non-dominant hemisphere for swallowing. Moreover, most studies of cerebellar TMS use conventional rTMS, which takes a long time and has high stimulus intensity, and few studies have used TBS.

In recent years, due to the advantages of non-invasiveness, high spatial and temporal resolution, and accurate positioning, resting-state functional magnetic resonance imaging (rs-fMRI) has become a hotspot in the research of human brain functions. Researchers often combine it with TMS to explore the neural mechanisms of the human brain (Bharath et al., 2015; Valchev et al., 2015). Among the measurements taken, the amplitude of low-frequency fluctuation (ALFF) is an indicator with good repeatability and definite physiological significance. By calculating the amplitude fluctuation in the blood oxygenation level-dependent (BOLD) signal deviating from the baseline (Zang et al., 2007), ALFF reflects the intensity of regional spontaneous neural activity in brain areas (Fox and Raichle, 2007). Increased ALFF indicates that spontaneous neural activity is active in local brain regions. This method has led to some achievements in the research of neural mechanisms in the field of swallowing (Ruan et al., 2017). Cerebellar iTBS has been shown to improve swallowing function in poststroke patients with dysphagia (Rao et al., 2022). Furthermore, the fractional amplitude of low-frequency fluctuation (fALFF) approach has high sensitivity and specificity in detecting spontaneous brain activities. However, to our knowledge, no study has used rs-fMRI to explore the effects induced by iTBS stimulating the cerebellum.

This study was performed with the aim of observing the effects of changes in spontaneous neural activity using iTBS on the dominant cerebellar hemisphere for swallowing and the non-dominant hemisphere for swallowing in healthy subjects and further exploring the mechanism of this intervention on swallowing-related brain areas. We hope this research can provide more theoretical basis for selecting new targets.

## 2. Materials and methods

### 2.1. Participants

Thirty-nine healthy subjects were recruited from July 2021 to March 2022, and all subjects signed informed consent for participation. This study was reviewed by the Ethics Committee of the Affiliated Hospital of Qingdao University. The subjects were divided by a random number table into three groups: the dominant cerebellum group, the non-dominant cerebellum group, and the sham group. A subject in the non-dominant cerebellum group withdrew from the study due to intolerance to cranial MR. Ultimately, data from 38 subjects were included in the final statistical analysis.

#### 2.1.1. Power calculations

Based on a 40% effect size which is suggested by previously published paper on cerebellar rTMS (Jayasekeran et al., 2011; Vasant et al., 2015), at least 12 healthy subjects would be needed for each group to achieve a power of 80% and statistical significance of 5% to demonstrate difference in effects among groups.

#### 2.1.2. Inclusion criteria

 (1) Age 18–40 years old.

 (2)  Is right-handed (assessed by the Edinburgh handedness inventory).

 (3) Has no significant past medical history.

 (4)  Volunteered to participate and be able to complete the experiment.

 (5) Signed informed consent in person.

#### 2.1.3. Exclusion criteria

 (1) Contraindications for MRI examination and TBS treatment or being unable to tolerate the TBS.

 (2) History of neuropsychiatric diseases or history of brain or otorhinolaryngologic surgery.

 (3) History of dysphagia, history of important organ diseases.

 (4) Use of anxiolytics, antidepressants or other drugs that affect the central nervous system, using psychotropic drugs.

 (5) Drug or alcohol abuse.

 (6) Current pregnancy.

### 2.2. Experimental methods

#### 2.2.1. Electromyography (EMG)

Subjects were seated, and surface electrodes were placed on the suprahyoid muscle surface to record EMG signals from the suprahyoid muscles. The recording electrode was placed 2 cm to the left and right of the midpoint of the line between the mandible and the middle part of the hyoid bone, the reference electrode was attached to the mandibular angle, and the ground electrode was placed on the lower forearm. All electrodes were connected to the EMG recording system (Yiruide, Wuhan, China).

#### 2.2.2. Single-pulse TMS

Cerebellar single-pulse TMS was performed using a CCY-IA TMS (Yiruide CCY-IA, Wuhan, China) with a circular coil (outer ring diameter: 70 mm) in the experiment, and the maximum stimulus intensity was 3.0 Tesla. Cerebellar single-pulse TMS was delivered by holding the coil upside down with its handle pointed superiorly.

#### 2.2.3. TBS stimulation protocol

Intermittent theta burst stimulation was performed over the “hot spot” of the cerebellar swallowing area identified by single-pulse TMS, and the coil was tangent to the scalp, with the handle pointing upward. The iTBS paradigm consisted of bursts of three pulses at 50 Hz repeated at 5 Hz. During iTBS, a 2 s train was followed by an 8 s intermission; this pattern was repeated 20 times for approximately 190 s (600 pulses). The iTBS was delivered at 100% resting motor threshold (rMT) (Rao et al., 2022).

### 2.3. Experimental protocols

Subjects were asked to participate in two trials with a 48-h intertrial interval (Sasegbon et al., 2020). In the first trial, single-pulse TMS was used to identify the “hot spots” in cerebellar representation of the suprahyoid muscles and to measure rMT. Then, the dominant cerebellar hemisphere for swallowing was determined. The dominant cerebellar hemisphere was defined as the hemisphere with a lower rMT. If the rMT was equal, the dominant cerebellar hemisphere with a higher baseline MEP was the dominant side (Dong et al., 2022). In this study, the rMT was measured as follows: first, using 60% output, coil movements were made in a region 1–3 cm below and 2–4 cm lateral to the occipital external carina. The region where the maximum MEP was obtained was the “hot spot” of the cerebellar representation of the suprahyoid muscles. Next, we applied single-pulse TMS on the hot spot to measure rMT. The rMT was defined as the minimum intensity of TMS that can evoke MEP amplitude greater than 50 μV in five out of a total of ten trials, expressed as a percentage of the stimulator’s maximum output intensity. When the bilateral rMT were equal, TMS was used to measure 5 times baseline MEP at the bilateral cerebellar hot spot, and the stimulation intensity was 100% rMT.

**FIGURE 1:**
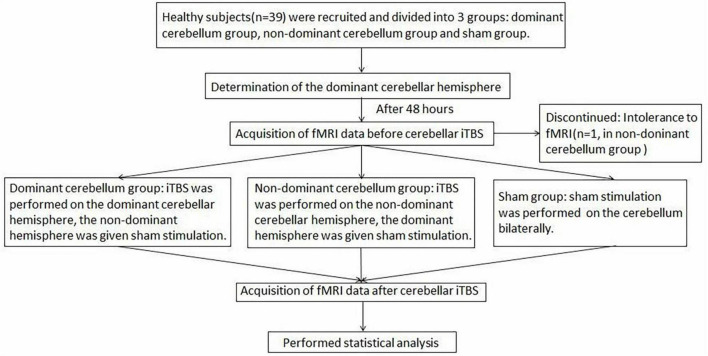
The study design and flow chart.

Since multiple uses of TMS when finding the “hot spot” and taking rMT measurements may affect cortical excitability, 48 h after elution, iTBS was applied to the cerebellum in the second trial. In the dominant cerebellum group, iTBS was administered to the dominant cerebellar hemisphere, and the non-dominant cerebellar hemisphere was given sham stimulation. In the non-dominant cerebellum group, iTBS was applied to the non-dominant cerebellar hemisphere, and sham stimulation was delivered to the dominant cerebellar hemisphere. In the sham group, sham stimulation was applied bilaterally to the cerebellum. Rs-fMRI was performed before and after TBS stimulation to observe changes in neural activity across the whole brain. Figure 1 shows the study design and flow chart.

### 2.4. Imaging data acquisition

Brain imaging was performed on a Signa HDX 3.0 T MRI scanner (GE Healthcare, Chicago, IL, USA). The participants were supine on the MRI scanner with their head movements restricted using foam padding and earplugs to reduce scanner noise. During scanning, participants were instructed to relax, breathe slowly and close their eyes but not to fall asleep. Blood oxygenation level-dependent signals were acquired by using a gradient echo single-shot echo-planar imaging sequence (30 axial slices, repetition time = 3,000 ms, echo time = 40 ms, flip angle = 90°, matrix = 96 × 96, inplane resolution of 1.875 mm × 1.875 mm, and thickness/gap = 5/0 mm). Subsequently, high-resolution T1-weighted images were obtained using a magnetization-prepared rapid acquisition gradient-echo (MPRAGE) 3-dimensional protocol (248 sagittal slices, repetition time = 3,000 ms, echo time = 3.9 ms, matrix = 256 × 256, and voxel size 1 mm × 1 mm × 1.2 mm).

### 2.5. Image data preprocessing

REST Plus V1.24 was used for resting-state fMRI image preprocessing as follows: Images were converted from DICOM format to Neuroimaging Informatics Technology Initiative (NIFTI) format. The first 10 volumes were removed. The remaining volumes were corrected for slice timing. Head motion correction (exclusion criteria were head motion exceeding 3 mm of movement or 2° rotation in any direction) was performed. The standard Montreal Neurological Institute (MNI) template provided by SPM12 was used for spatial normalization and data were resampled with a voxel size of 3 mm × 3 mm × 3 mm. The images were smoothed using a 4 × 4 × 4 kernel in SPM software version 12 Linear detrending. The white matter, cerebral spinal fluid signal and the Friston 24 head-motion parameters were removed by regression. RESTplus v1.82 software was used to calculate the Z-standardized fractional amplitude of low-frequency fluctuation (zfALFF) values and generate the zfALFF maps. Using the software package RESTplus v1.24, images of subjects who received left cerebellar stimulation were flipped so that the stimulation side of all subjects was uniformly on the right.

### 2.6. Statistical analysis

SPSS 26.0 was used to compare the demographics of the three groups. The chi-square test was performed to analyze categorical data, and one-way analysis of variance (ANOVA) was used for continuous variables. *P* < 0.05 was considered to indicate statistical significance.

The DPABI V5.1 software package was used for rs-fMRI analysis. Paired-sample *t*-tests were used to determine significant differences between baseline and after iTBS in the fALFF maps for each group. One-way ANOVA was performed to compare whole-brain fALFF maps of the three groups before iTBS. Then, in the same way, we compared fALFF maps of the three groups after stimulation. In addition, *post-hoc t*-tests were applied to examine the differences of fALFF values between groups. The resulting statistical map was set at a threshold of *P* = 0.05 (uncorrected) and a minimum cluster size of 100 voxels.

## 3. Results

### 3.1. Participants

There were no significant differences in sex or age among the three groups, as shown in Table 1. In 15 of the 38 subjects, the dominant cerebellar hemisphere was on the left side, whereas in the remainder, the dominant cerebellar hemisphere was on the right side.

**TABLE 1:** Demographic information of subjects.

	Sex (male/female)		Dominant side (left/right)		Age (mean ± SD years)
Dominant cerebellum group (*n* = 13)	7	6	3	10	24.31 ± 3.38
Non-dominant cerebellum group (*n* = 12)	7	5	6	6	26.75 ± 3.19
Sham group (*n* = 13)	7	6	6	7	24.77 ± 2.52
*p*-value	0.967^a^		0.323^a^		0.122^b^

^a^Indicate the *p*-value for the Chi-Square test; ^b^indicate the *P*-value for the ANOVA F-test.

### 3.2. Comparison of fALFF before iTBS among the three groups

There was no statistically significant difference in fALFF before iTBS among the three groups.

### 3.3. Comparison of fALFF between post-iTBS and pre-iTBS in each group

**FIGURE 2:**
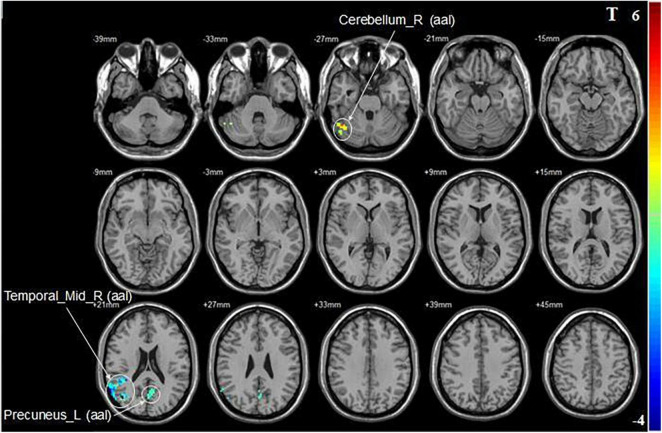
Statistical maps showing fALFF change pre- and post-iTBS in the dominant cerebellum group. Warm colors showing fALFF increased and cool colors showing fALFF decreased after iTBS.

Compared with pre-iTBS, stimulation of the dominant cerebellar hemisphere showed an increase in fALFF in the ipsilateral cerebellum, and decreased fALFF in the ipsilateral middle temporal gyrus, and contralateral precuneus (Figure 2 and Table 2).

**TABLE 2:** Brain regions with alteration of fALFF after iTBS in different groups.

Brain region	Cluster size (voxel)	Coordinates (*x*, *y*, *z*)	Peak *T*-value
**Dominant cerebellum group**			
Cerebellum_Crus1_R (aal)	165	51, −48, −27	5.4275
Temporal_Mid_R (aal)	162	42, −72, 21	−5.003
Precuneus_L (aal)	141	3, −69, 21	−4.043
**Non-dominant cerebellum group**			
Frontal_Sup_2_R (aal)	127	21, 24, 51	10.7939
Calcarine_R (aal)	357	33, −66, 33	8.5007
Parietal_Inf_L (aal)	168	−51, −66, 39	8.0398

aal, anatomical automatic labeling; L, left; R, right; T, statistical value of peak voxel showing fALFF changes pre- and post-iTBS (negative values: fALFF decreased after iTBS; positive values: fALFF increased after iTBS).

**FIGURE 3:**
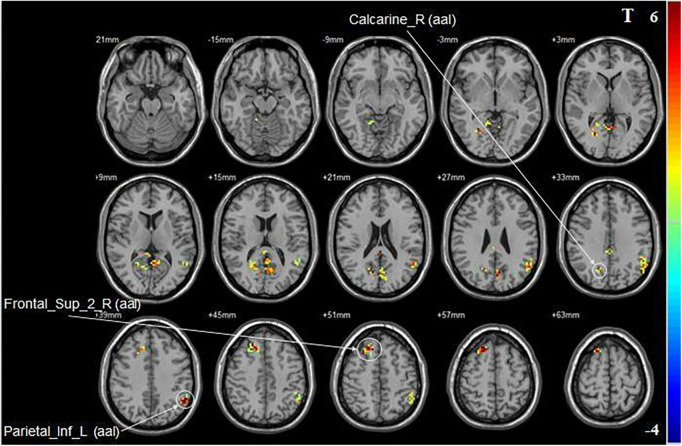
Statistical maps showing fALFF change pre- and post-iTBS in the non-dominant cerebellum group. Warm colors showing fALFF increased and cool colors showing fALFF decreased after iTBS.

Compared with baseline, iTBS applied to the non-dominant cerebellar hemisphere induced increased fALFF in the ipsilateral superior frontal gyrus, the calcarine fissure and surrounding cortex, and the contralateral inferior parietal lobule (Figure 3 and Table 2).

In the sham group, there was no significant difference in fALFF between baseline and after intervention.

### 3.4. Comparison of fALFF after iTBS among the three groups

**FIGURE 4:**
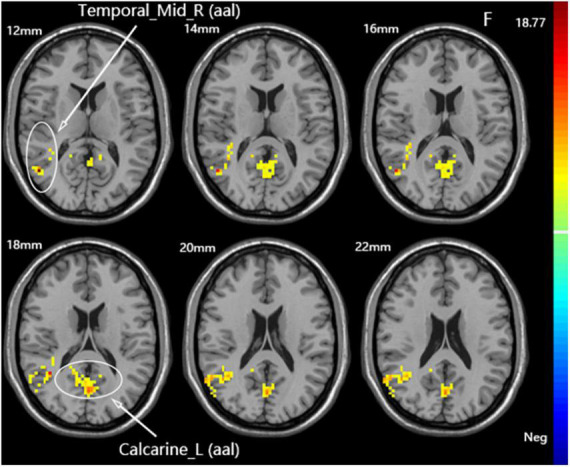
Statistical maps showing fALFF difference after iTBS among the three groups. The color bar indicated the *F*-values of the ANOVA analysis.

A one-way ANOVA revealed significant differences in fALFF among the three groups in the ipsilateral middle temporal gyrus and contralateral calcarine fissure and surrounding cortex (Figure 4 and Table 3).

**TABLE 3:** Brain regions with fALFF differences after iTBS in dominant cerebellum group, non-dominant cerebellum group, and sham group.

Brain region	Cluster size (voxel)	Coordinates (*x*, *y*, *z*)	Peak *F/T*-value
**Differences among three groups**			
Calcarine_L (aal)	207	0, −69, 18	10.0172
Temporal_Mid_R (aal)	104	51, −63, 12	18.7688
**Dominant vs. sham group**			
Calcarine_L (aal)	185	0, −69, 18	−4.1367
**Non-dominant vs. sham group**			
No difference	–	–	–
**Dominant vs. non-dominant group**			
No difference	–	–	–

Dominant, the dominant cerebellum group; Non-dominant, the non-dominant cerebellum group; sham, the sham group; aal, anatomical automatic labeling; L, left; R, right; F/T, F/T-statistical value of peak voxel showing fALFF differences among groups.

**FIGURE 5:**
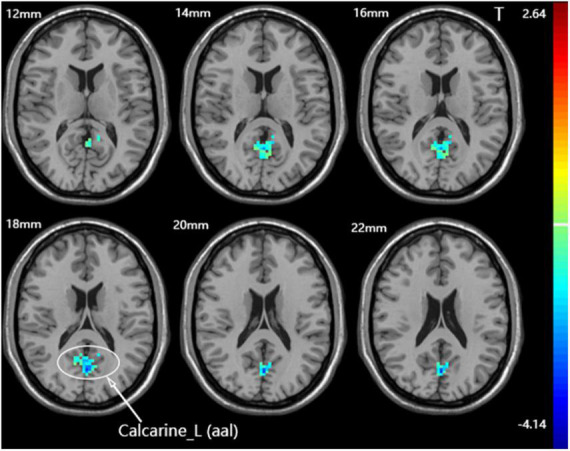
Statistical maps showing fALFF difference after iTBS between the dominant cerebellum group and the sham group. Warm colors showing fALFF increased and cool colors showing fALFF decreased.

Compared with the sham group, the dominant cerebellum group had decreased fALFF in the contralateral calcarine fissure and surrounding cortex (Figure 5 and Table 3). No statistically significant difference in fALFF between dominant cerebellum group and non-dominant cerebellum group. The same result was observed between non-dominant cerebellum group and sham group.

## 4. Discussion

### 4.1. Comparison of fALFF between post-iTBS and baseline

Swallowing is a complex sensorimotor behavior involving the regulation of extensive neural networks. Regions from the cerebral cortex to subcortical structures, brain stem, and cerebellum are involved in the control of swallowing (Ertekin and Aydogdu, 2003). Functional brain imaging studies of humans have shown that the cerebral cortical centers involved in swallowing regulation are multi-centered and are mainly located in the sensory/motor cortex, prefrontal cortex, anterior cingulate gyrus, insula, parietooccipital region, temporal lobe, and other regions (Hamdy et al., 1999a,b; Mosier et al., 1999; Zald and Pardo, 1999). The basal ganglia, thalamus, cerebellum, and other areas also play vital roles in swallowing (Mosier et al., 1999). However, the specific functions of brain regions and their connectivity are still not clear. At present, the effect of cerebellar rTMS on the swallowing motor area of the cerebral cortex has been relatively explicit. A number of previous studies used electrophysiological techniques to demonstrate the effect that unilateral cerebellar rTMS can excite swallowing motor cortex of bilateral cerebral hemispheres, and its neural mechanism were also explored (Sasegbon et al., 2019, 2020). Cerebellar rTMS is able to cause the excitation of swallowing motor cortex of contralateral cerebral hemisphere through the cerebello-thalamo-cortical connections (Daskalakis et al., 2004; Roostaei et al., 2014). And there may be two reasons to explain why unilateral cerebellar rTMS is able to provoke the excitation of ipsilateral cerebral hemisphere. The cerebellar fastigial nucleus send projections to the nucleus ambiguus in the medulla, which is part of the central pattern generator (CPG). As subcortical swallowing center, CPG is connected with the swallowing centers in the bilateral cerebral cortex. This pathway provides a potential explanation for the ipsilateral effect (Jean, 2001; Zhang et al., 2016; Sasegbon and Hamdy, 2017). In addition, interhemispheric communication may result in the ipsilateral effect (Takeuchi et al., 2012). However, the effects and mechanisms of cerebellar rTMS on other brain regions related to swallowing are still unclear. Now, we hope to fill the gap of knowledge in this field by using resting-state functional magnetic resonance imaging.

In our study, we applied iTBS to the dominant cerebellar hemisphere, and found increased fALFF in the ipsilateral cerebellum, indicating that iTBS facilitated the targeted area, resulting in enhanced spontaneous neural activity. The main role of the cerebellum is to control and monitor motor behavior. The cerebellum regulates motor commands by integrating proprioception, somatosensory, vision and vestibular sensation, and coordinates the movement direction, time and strength of different muscle groups (Rangarathnam et al., 2014) to ensure that the voluntary movement of limbs can be coordinated, accurate, and smooth (Roostaei et al., 2014). With the rapid development of functional imaging, a large number of functional neuroimaging studies have shown that the cerebellum is activated in a variety of swallow-related movements, such as chewing (Onozuka et al., 2002), lip and tongue movements (Grodd et al., 2001), and orofacial movements (Sörös et al., 2006). These findings confirmed that the cerebellum plays an indispensable role in swallowing movement, which has prompted researchers to further explore the function of the cerebellum in swallowing regulation. Cerebellar mapping studies have shown that the representative areas of the lips and tongue are located in the cerebellar hemispheres and vermis (Boillat et al., 2020), which provides strong evidence for the presence of cerebellar swallowing motor areas. In addition, Mosier and Bereznaya (2001) proposed the theory that swallowing function occurs due to the activation of parallel networks composed of five functional modules by analyzing the functional brain maps of swallowing tasks in healthy people through structural equation modeling. Among them, the cerebellum, as an independent module, may play a key role in the time regulation, internal coordination of oropharyngeal muscles and feed-forward mechanisms. Moreover, the cerebellum was activated during lip, tongue, and jaw movements and vowel vocalization in healthy people. As the key point of the core functional motor network controlling laryngeal and supralaryngeal movements, the cerebellum is considered to mainly receive motor and sensory information and participate in fine muscle coordination of movements (Grabski et al., 2012). We speculated that stimulation of the dominant hemisphere of the cerebellum may promote the coordination and fluency of swallowing movement and increase the accuracy of swallowing movement mainly by facilitating the target cerebellum.

We also found that after iTBS application over the dominant cerebellar hemisphere, fALFF values were decreased in the ipsilateral middle temporal gyrus and the contralateral precuneus. The temporal lobe is associated with a variety of swallowing functions: it is involved in human taste processing and recognition of gustatory stimuli (Small et al., 1997). In addition, due to their close association with the taste and imagery of food, the temporal cortex and prefrontal cortex play an auxiliary role in regulating swallowing (Hamdy et al., 1999a). The posterior parietal cortex is the hub for integrating sensory input with motor output (Kern et al., 2001). As part of the posterior parietal cortex, the precuneus is involved in the reception and advanced processing of oropharyngeal, and esophageal sensation (Kaderali et al., 2015). In this group, iTBS resulted in decreased neural activity in the contralateral precuneus, which laterally verified the findings of Casula et al. (2016). In that study, TMS-EEG technology was used to evaluate the effect of cerebellar iTBS on the cerebral cortex in healthy subjects. It was found that cerebellar iTBS reduced neural activity in the contralateral posterior parietal cortex. Moreover, by using structural equation modeling (SEM) to analyze functional brain maps for swallowing tasks in healthy adults, Mosier and Bereznaya (2001) pointed out that volitional swallowing movement occurs through the activation of a parallel network composed of five functional clusters or modules: (1) sensorimotor areas and cingulate gyrus; (2) inferior frontal gyrus, S2, corpus callosum, basal ganglia and thalamus; (3) premotor cortex and posterior parietal cortex; (4) cerebellum; and (5) insula. Each module performs certain functions independently during swallowing. At the same time, they are functionally connected to other modules and form a functional network. Among them, the cerebellum has a negative influence on multiple modules of the cerebral cortex, and facilitates the coordination of swallowing movement through functional (but not anatomical) connectivity. We infer that iTBS significantly increased the excitability of the dominant cerebellar hemisphere, which may decrease the spontaneous neural activity of the bilateral cerebral cortex through negative regulation effect.

After iTBS stimulated the non-dominant cerebellar hemisphere, increased fALFF was found at the ipsilateral superior frontal gyrus, the calcarine fissure and surrounding cortex, and the contralateral inferior parietal lobule. The superior frontal gyrus is involved in motor planning of consecutive movements such as swallowing (Haupage et al., 2010), and the prefrontal lobe is related to the perception of food flavor and taste (Lee et al., 2018) and the integration of sensorimotor information (Suntrup et al., 2014). The calcarine fissure and surrounding cortex are part of the occipitoparietal region, which is widely activated in swallowing and swallowing-related motor tasks in healthy people (Kern et al., 2001). As a general hub, the occipitoparietal region is mainly involved in the reception and further processing of sensation (Ertekin and Aydogdu, 2003), as well as the association of visual and auditory input to sensorimotor responses (Kern et al., 2001). Swallowing water was linked to the activation of the inferior parietal lobule, which may reflect multiple sensory processing when water stimulated the mouth, such as the temperature and gustatory (Sörös et al., 2009). In addition, the functional connectivity between the inferior parietal lobule and insular lobes is regulated by visceral sensory stimulation (Babaei et al., 2013), both of which are important components of the brain swallowing network. iTBS of the non-dominant cerebellar hemisphere mainly increased the excitability of multiple brain regions of the bilateral cerebral cortex, which may contribute to the reception and processing of sensation and the integration of sensorimotor information during swallowing. Under the static state, the dominant cerebellar hemisphere plays a leading role in regulating swallowing (the negative effect on cerebral cortex). Although the “silent” non-dominant cerebellar hemisphere didn’t show a statistically significant increase of spontaneous neural activity when stimulated by exogenous stimulus, we speculated that the intrinsic excitability of the non-dominant cerebellar hemisphere may enhanced, thereby weaken the negative regulatory effect of the dominant cerebellar hemisphere on the cerebral cortex, resulting in the spontaneous neural activity in several swallowing-related brain regions of cerebral cortex enhanced than before stimulation.

Finally, there was no statistically significant difference in the sham group between baseline and after intervention, which was consistent with our prediction.

### 4.2. Comparison of fALFF after iTBS among the three groups

We also compared the fALFF after iTBS among the three groups. After iTBS, the fALFF values of the ipsilateral middle temporal gyrus and the contralateral calcarine fissure and surrounding cortex were statistically different among the three groups. In subsequent pairwise comparisons, we found decreased fALFF in the contralateral calcarine fissure and surrounding cortex in the dominant cerebellum group compared with the sham group. This phenomenon is similar to the inhibitory effect of cerebellar TMS on the contralateral cerebral motor cortex (cerebellar brain inhibition) (Ugawa et al., 1995). As for the ipsilateral middle temporal gyrus, the one-way ANOVA showed the fALFF values of this brain region were different among the three groups, but there was no statistical difference in the pairwise comparisons between groups, which may be related to the fact that the minimum cluster size we set in the statistical analysis was not less than 100 voxels.

There were some limitations in our study. First, the subjects participating in this study were all young people and the sample size was relatively small, which resulted in a lack of representative data. In the future, we will conduct experiments in different age groups, and more subjects should be recruited to verify the current findings. Second, all subjects in our study were healthy people, and the effect of iTBS stimulation on stroke patients cannot be directly simulated. We will improve the experimental protocol to observe the effects of iTBS with different stimulation parameters on patients with poststroke dysphagia and further explore the neural mechanism of iTBS in improving swallowing function.

## 5. Conclusion

In this study, we found that iTBS of the dominant cerebellar hemisphere for swallowing excited the ipsilateral cerebellum, and caused transient inhibition of some cerebrocortical areas. Stimulation of the non-dominant cerebellar hemisphere increased the spontaneous neural activity of multiple cerebrocortical areas related to swallowing. In conclusion, regardless of which side of the cerebellum is stimulated, iTBS can facilitate part of the brain neural network related to swallowing. Our findings reveal the short-term effect of cerebellar iTBS on spontaneous brain activation, and provide supporting evidence that cerebellar iTBS can be used as a potential method to modulate human swallowing movement.

## Data availability statement

The original contributions presented in this study are included in the article/supplementary material, further inquiries can be directed to the corresponding authors.

## Ethics statement

The studies involving human participants were reviewed and approved by the Ethics Committee of the Affiliated Hospital of Qingdao University. The patients/participants provided their written informed consent to participate in this study.

## Author contributions

BW, QW, and PM contributed to the conception and design of the study. WM collected the data. XP and LD performed the statistical analysis. BW wrote the first draft of the manuscript. QW, PM, HS, and BW wrote sections of the manuscript. All authors contributed to manuscript revision, read, and approved the submitted version.
