# Corrigendum: The effects of intermittent theta burst stimulation of the unilateral cerebellar hemisphere on swallowing-related brain regions in healthy subjects

**DOI:** 10.3389/fnhum.2023.1217476

**Published:** 2023-05-23

**Authors:** Bingyan Wang, Hui Sun, Xiaona Pan, Wenshuai Ma, Linghui Dong, Qiang Wang, Pingping Meng

**Affiliations:** ^1^Department of Rehabilitation Medicine, Affiliated Hospital of Qingdao University, Qingdao, China; ^2^Department of Radiology, Affiliated Hospital of Qingdao University, Qingdao, China

**Keywords:** swallowing, cerebellum, intermittent theta burst stimulation (iTBS), resting-state functional magnetic resonance imaging (rs-fMRI), fractional amplitude of low-frequency fluctuation (fALFF)

In the published article, there was an error in the **Funding** statement. We found a funding “Qingdao Outstanding Health Professional Development Fund” that we forgot to add. The original text is “This work was supported by the Key Research and Development Projects of Shandong Province (2019GSF108262) and Shandong Provincial Natural Science Foundation (ZR2020MH282).” The correct **Funding** statement appears below.

